# Vaccine Trends in Pakistan: A Review of Immunization Challenges and Setbacks Prompted by Inadequate Disaster Management

**DOI:** 10.7759/cureus.55357

**Published:** 2024-03-01

**Authors:** Angelina Zarzeczny, Payal Kahar

**Affiliations:** 1 Public Health, Florida Gulf Coast University, Fort Myers, USA; 2 Health Sciences, Florida Gulf Coast University, Fort Myers, USA

**Keywords:** immunization, covid-19, vaccine-preventable diseases, emergency response, vaccination initiatives, disaster management, immunization coverage, pakistan

## Abstract

Pakistan bears an incredible burden of vaccine-preventable diseases, and efforts to improve widespread immunization have been hindered by inadequate public health management following disasters and other health emergencies. Through a brief review of Pakistan’s health system, an understanding of routine immunization challenges is sought based on the organizational changes made to the planning and delivery of immunization activities. Further, recent immunization trends of measles, polio, and tuberculosis are examined in correspondence to health emergencies prompted by climate change and the COVID-19 pandemic. The national public health response to each disease is discussed, and insight is given to how the devolution of Pakistan’s health system may have influenced the severity of each emergency. Focus is given to the potential immunization challenges and how they may impact future initiatives for the control of vaccine-preventable diseases. Although incidence rates suggest increased cases of certain vaccine-preventable diseases and disruptions of immunization activities following recent disasters, further studies may need to be conducted to establish a stronger understanding of the immunization trends noted within this review.

## Introduction and background

Pakistan has recently been a focus of global attention due to its struggle in polio eradication, yet the country’s challenges are further exemplified by its poor management of routine immunization during times of health emergencies. With a cumulative average national vaccination rate of 60.6% and one of the highest morbidity and mortality rates in children less than five years old, vaccine-preventable deaths constitute nearly half of all deaths in Pakistan [1]. Several factors may be responsible for decreased coverage, such as household income, education, and degree of rurality, yet these factors do not explain why coverage has decreased following initial improvements to immunization coverage. In fact, in 1978, Pakistan made a major advancement towards improving routine immunization through the creation of the Expanded Programme on Immunization (EPI). Generally, the EPI aims to reduce childhood morbidity and mortality through widespread immunization of childhood tuberculosis, poliomyelitis, measles, and other vaccine-preventable diseases (VPDs), and recent initiatives plan to reduce the infant mortality rate from 74% to less than 40% by 2025 [2,3]. Currently, the EPI’s goals for measles and polio eradication in Pakistan have yet to be achieved, but the country has made striking improvements in immunization status since the program’s founding. Furthermore, Pakistan’s Demographic and Health Survey for 1990-1991 revealed that in children between 12 and 23 months old, 70% received a BCG vaccine, 50% a measles vaccine, and 43% were fully immunized against polio [4]. In 2006-2007, the survey depicted further improvement in immunization status as the vaccination rates were 80%, 60%, and 93% for BCG, measles, and a single dose of polio, respectively [5]. Yet despite this increasing trend, Pakistan has appeared to fall behind in immunization coverage in times of emergency, which may have been exemplified by the decentralization of Pakistan’s government in 2010 [2,3]. In the past, Pakistan’s Federal Ministry of Health was primarily responsible for planning and distributing health services, while provincial governments held a passive influence [2]. However, these roles reversed in 2010 when the central government dispersed more power to provinces and loosened control over health decisions [6]. Instead of having responsibilities tied to a single source of authority, a decentralized system formed several smaller points of power that shared many interconnections. Likewise, following the decentralization of Pakistan’s government, responsibilities for health services and planning were delegated to regional providences, yet despite embracing a more bottom-up targeted approach, the success of delegated duties is hindered by unclearly defined roles in health programs and interventions [2]. Furthermore, the federal EPI implements national health policies and remains responsible for matters such as purchasing vaccines, but poor clarification in policies and a lack of inter-provincial coordination have led to inequitable service delivery on behalf of the provincial governments [7]. For example, the EPI policy specifies the number of vaccinators required but does not offer clarification on additional details which has contributed to inconsistencies in training, monitoring, and proper capacity-building [7]. Not only that but in a study of provincial EPI managers in Pakistan, many noted that a major challenge to immunization was the lack of up-to-date surveillance data [7]. The study participants claimed that population estimates and estimates on vaccines and staff were based on population data from a 1998 census. Between inaccurate population estimates and a lack of coordination among provinces, children are likely to be missed during immunization campaigns, and the true representation of an immunized population in Pakistan remains unclear.

While delegating powers to local governments is often useful in understanding the specific needs of the community, it fails to adequately deliver services when there are no clear roles defined for each government tier. Given this, health programs and interventions that were previously implemented and regulated by the Federal Ministry of Health may not have been carried out as effectively without a structural backbone to health policy. For instance, 10 vertical programs, including EPI, Roll Back Malaria, and TB-DOTs, were in effect prior to devolution but later faced challenges such as fragmented service delivery, duplicated resources, and delayed fund releases due to centralized budgets [6]. Under provincial regulation, these programs demonstrated inconsistent delivery as a result of finances still being largely centralized even after devolution. Allocating a decentralized budget may be easier if these programs were integrated into the health system itself, however, integration is often resisted by provincial vertical program managers [6]. As an integrated system, vertical programs like national immunization days and vaccine drives could greatly improve the impact of routine immunizations, but isolated and with limited funding, the effectiveness of these programs degrade. All in all, several studies have examined Pakistan’s immunization trends in relation to one or more VPDs, but to the authors’ knowledge, this is the first review to suggest decentralization as a key factor in decreased immunization rates of VPDs during disasters. Furthermore, the aim of this review is to examine the immunization challenges posed by unprecedented health emergencies, such as the COVID-19 pandemic and seasonal flooding, and how they have highlighted the need for a more consistent health policy structure between the provincial health sectors. Moreover, routine immunizations are an integral component of a sustainable health system, and vaccine administration in Pakistan may continue to fall behind in immunization coverage if focus is not diverted to fixing organizational instability and improving disaster preparedness.

## Review

Methods

Articles for this review were searched through Google Scholar and PubMed with publication dates restricted between 2010 and 2023. Furthermore, keywords such as “Pakistan,” “vaccine preventable,” “immunization,” and “disaster” were used interchangeably as search terms. Other terms like “COVID-19” and “floods” were also used to produce more relevant results. Articles that were not in English or available in full text were not considered. For the purpose of this review, only articles that specifically referred to the immunization of VPDs were considered for screening. That said, 58 articles were screened, and the reference lists of these articles were screened as well. Overall, a total of 15 articles were deemed relevant and included in this review.

Routine immunization collaborations and financing

Decentralization has posed new challenges for vaccine administration in Pakistan, but through collaborations with vaccinators and community health workers, such as Lady Health Workers, Pakistan has been able to improve immunization outreach and resolve delegation-based issues that arose from the shift in health responsibilities. Although there is often little to no formal training for vaccinators in Pakistan, they are often able to effectively manage duties with the aid of community workers who are trusted by and familiar with the population. Since most of the Pakistani population is likely familiar with routine immunization through door-to-door campaigns, such as those that were held for polio, the Lady Health Workers (LHWs) Program is usually an effective form of outreach, especially in rural and underdeveloped areas [6,8]. As a matter of fact, studies have shown that since the implementation of LHWs, served areas have demonstrated significantly better health indicators, and the greatest factor in incomplete immunization of infants was the absence of LHWs in the community [8]. As a cost-effective public health intervention, LHWs have not only significantly improved childhood immunization against communicable diseases but have illustrated how community-based interventions may be more effective than broad, overarching interventions that do not utilize community relationships.

Yet partnerships often do not come without finances, and Pakistan’s low health expenditure and heavy dependence on international donors pose several challenges to immunization progress. Despite a fairly large increase in proportionate spending on health in the provincial sectors, decentralization also created delays in federal health transfers that serve as a key funding source for vertical programs and LHWs [6]. Furthermore, only 0.7% of Pakistan’s GDP is spent on health, and approximately two-thirds of preventative program support comes from international donors [6,9]. As a Tier 1 priority country, Pakistan is one of the largest recipients of Global Alliance for Vaccines and Immunization (GAVI) funding, and the majority of the aid is used to purchase vaccines and support vertical programs [2]. Provincial governments, on the other hand, are responsible for EPI staff salaries, supplies, transportation, and maintenance [6]. However, in order to use this funding, budget approvals must first be approved by the central legislature before moving to the provincial health departments, in which the District Health Officer must release the funds to EPI [6]. Since finances are not immediately received by the EPI, there is often a delayed release of funding that could be avoided if there was a separate budget line for the EPI altogether [6]. This would allow for the development of a more efficient budget while also allowing for the rapid procurement and distribution of vaccines. While awaiting budget approval, the purchasing of vaccines may be delayed and the supply may not be sufficient, resulting in possible missed vaccinations. With that said, external assistance from organizations like GAVI has allowed Pakistan to improve its national immunization coverage, but under health emergencies, weak routine immunization coverage has pushed Pakistan towards surges of several VPDs.

Immunization challenges

Most of the issues with the management of routine immunizations during health emergencies tie into a lack of proper preparedness and delegation of duties following the decentralization of Pakistan’s health system. Utilizing a bottom-up approach to increase authority to provincial governments helped community-based efforts like the LHWs become more organized and effective, but improper planning still makes it difficult for Pakistan to provide routine immunizations in marginalized populations and rural areas in times of crisis. Furthermore, the percentage of children immunized is typically unreliable because of inconsistent record-keeping practices and poor disease surveillance systems [2]. In addition, while polio uses the EPI workforce yet has its own administration, financing, and reporting systems [6], diseases like tuberculosis and measles fall short of prioritization because they are typically managed with routine immunization as a whole. Not only that, but disease trends have shown that much of the Pakistani population fails to recognize the significance of routine immunization due to a lack of accessible health information but also the promotion of vaccinations. Given that the strongest pushes for immunization came with polio eradication and later the COVID-19 pandemic, advocacy for other vaccinations does not come easily when people begin to question why their importance was only brought up suddenly. Perhaps if Pakistan engaged the private sector of health, or often the first contact of care, with the promotion of routine immunizations [2], Pakistan could improve the acceptance and delivery of vaccines for polio, tuberculosis, and measles. However, vaccine acceptance and surveillance are only part of the approach necessary to improve routine immunization as Pakistan is inadequately prepared to continue basic health activities during an emergency.

Pakistan is frequently prone to flooding as a result of excessive rainfall, and while the country has enacted measures to mitigate impacts on infrastructure, an insufficient disaster risk reduction (DRR) system and displacement of populations contribute to disruptions in health service delivery. Not only has Pakistan’s DRR system notedly suffered from unclear agendas and a lack of political commitment, but flood mitigation efforts have been made without a comprehensive plan that would indicate whether the actions were targeting the country’s greatest points of vulnerability during a flood [10]. Given this, the delivery of immunizations and basic health services have been affected by recent floods that have severely impacted the general population and challenged Pakistan’s resources for disaster management. During the 2022 floods, for example, over 600,000 individuals were displaced and 2,000 health facilities were impacted [11]. Likewise, damaged bridges and roads as well as shortages in medicine and equipment also impacted the accessibility, availability, and quality of health services [12]. Yet, even though many providences likely shared damage to their health infrastructure, two of the providences most affected by the floods, Balochistan and Sindh, displayed some of the lowest proportions of immunized children with immunization coverages of 37.5% and 49%, respectively [12]. In response, the WHO deployed 2,500 vaccinators to deliver routine immunizations in flooded areas of Sindh, but challenges remained with routine immunizations for flood-affected areas [13]. In fact, Dr. Baseer Khan Achakzai, the Director General of the Ministry of National Health Services Coordination and Regulations, stated in an interview that the displacement of immunization records and destruction of about 1,700 vaccination sites decreased immunization coverage to 64% after the 2022 floods [14]. These challenges, however, could have been reduced if DRR strategies required all health facilities to use electronic rather than paper immunization records [7] and maintain an adequate supply of medicine and vaccines. Simply put, Pakistan’s experience with seasonal flooding has demonstrated that although the true impact of an emergency may be unpredictable, the extent of future disasters may be reduced if Pakistan employs a proactive response to events that could affect routine immunizations rather than being reactive to health issues as they arise.

Effect of seasonal rain and floods on measles

**Figure 1:**
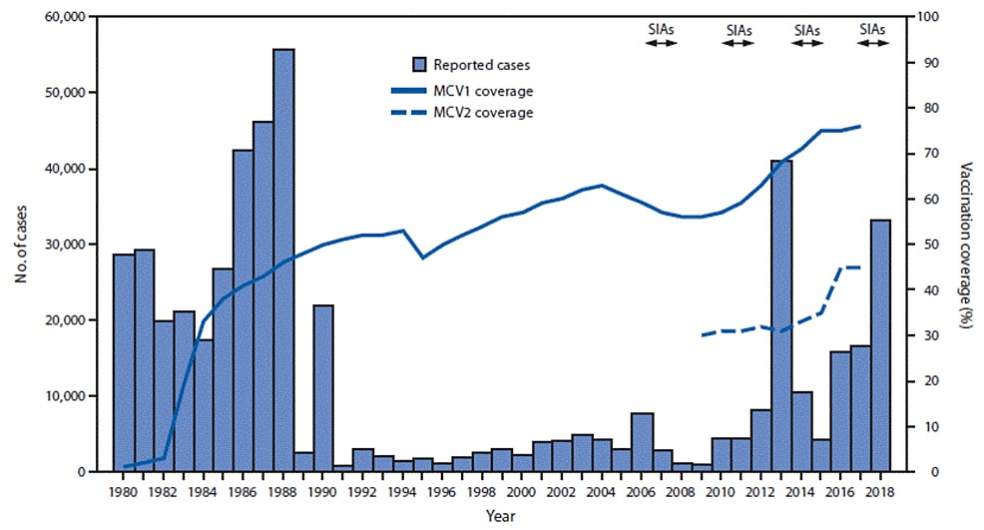
Number of reported measles cases and estimated coverage with the first and second doses of measles-containing vaccine (MCV), and supplemental immunization activities (SIAs), by year — Pakistan, 1980–2018 *Figure obtained from the Centers for Disease Control and Prevention [16] in the public domain.

Climate change has prompted the occurrence of natural disasters across the world, and with excessive rainfall and monsoon conditions, some of Pakistan’s most flood-prone areas have experienced an increase in infectious diseases. Furthermore, in 2010, Pakistan experienced a flood of unprecedented severity that had a notable impact on the incidence of measles following the displacement of 46.9% and forced migration of 86.8% of families [15]. Likewise, Figure 1 depicts a dramatic increase in reported measles cases with under 5,000 in 2010 and over 40,000 in 2013 [16]. Although the flood occurred three years prior to the observed increase, this could be due to disruptions in reporting caused by damaged infrastructure and poor surveillance. Additionally, Figure 1 displays a potential correlation between an increase in cases and a drop in measles-containing vaccine (MCV) coverage marked in 2010 [16]. The historic flood likely contributed to decreased immunization coverage due to inaccessible vaccination sites, destroyed medical records, and disrupted supplementary immunization activities (SIAs). Yet, this trend is not a singular occurrence as Figure 1 depicts a marked increase in measles cases to about 33,000 in 2018 corresponding to severe monsoon activity near Punjab [16,17]. Given this, over one-third of the country, or approximately 33 million people, experience severe flooding or monsoons every year, and while these events have been associated with increases in various diseases, the risk of measles does not share the same recognition [18].

Excessive rainfall has heightened the severity and occurrence of floods in Pakistan, yet the significance of Measles, Mumps, and Rubella (MMR) vaccination is undermined. Despite each disease being vaccine-preventable, Pakistan experienced a 65% decline in MMR vaccination and had the sixth most global cases of measles with 6,199 in January 2024 [18-20]. Although not all floods may compare to one Pakistan encountered in 2010, recent natural disasters may exhaust what little resources are left after the COVID-19 pandemic. Furthermore, as an estimated 40 million children remain unvaccinated against measles in 2020, the displacement of families, overridden hospitals, and poor sanitary conditions after flooding may worsen the measles epidemic in Pakistan [20]. In general, public health perceives water, sanitation, and hygiene as the first needs to be addressed after a severe flood because families may acquire various diarrheal and infectious diseases from poor hygienic conditions in displacement shelters. However, floods also indirectly affect health by blocking roads to access health services and potentially destroying medical records. Since Pakistan often still uses paper records at health facilities [6], information is more likely to become destroyed or misplaced in the case of a flood, so MMR routine immunization data and reported cases of measles may be inaccurate. As one of the most contagious diseases, measles outbreaks are just one of many VPDs that may spike after flooding because Pakistan is unable to prioritize routine immunizations equally with emerging health problems.

Impact of COVID-19 on poliomyelitis

**Figure 2:**
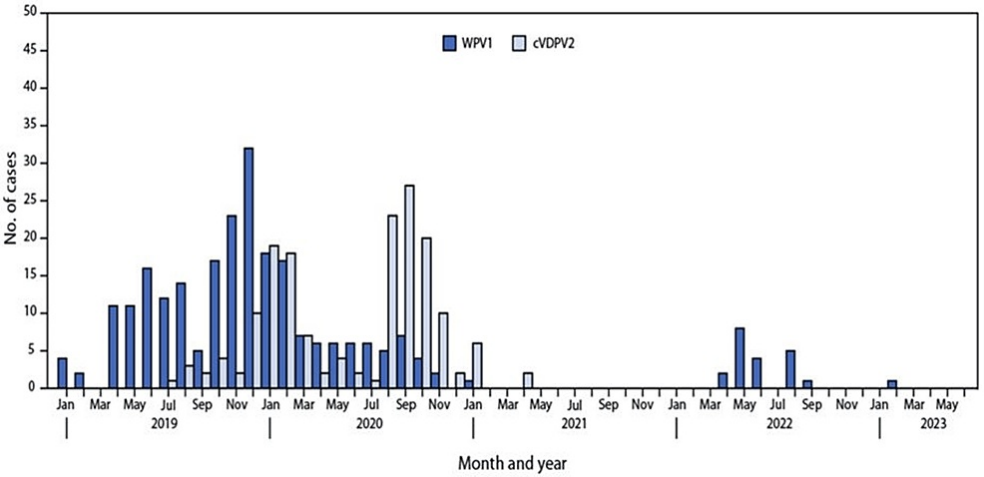
Wild poliovirus type 1 and circulating vaccine-derived poliovirus type 2 cases, by month — Pakistan, January 2019–June 2023 Abbreviations: cVDPV2 = circulating vaccine-derived poliovirus type 2; WPV1 = wild poliovirus type 1. *Figure obtained from the Centers for Disease Control and Prevention [23] in the public domain.

Since the country already struggled with a low vaccination coverage rate, the unprecedented COVID-19 pandemic exacerbated many of the flaws that were already present in the routine immunization system and created new challenges Pakistan may not be able to readily address. After many years of door-to-door campaigns for most routine immunizations, people expected all immunizations to be delivered in the same fashion, but the pandemic limited LHW outreach and discouraged people from seeking out vaccinations [6]. Not only did mandatory lockdown policies restrict visits from vaccinators, especially in remote areas, but the fear of exposing themselves to COVID-19 in hospitals and vaccine misinformation all hindered routine immunization progress [21]. For example, as one of the only countries polio endemic in 2023, disruptions in routine immunizations were detrimental to eradication progress as it resulted in an estimated 50 million children missing their polio vaccinations [20,22]. These missed vaccinations may have a detrimental impact on Pakistan’s polio eradication progress, particularly due to the interesting pattern of wild poliovirus 1 (WPV1) and circulating vaccine-derived poliovirus 2 (cVDPV2) cases displayed in Figure 2. Before the pandemic, 147 WPV1 cases were reported in 2019 with few cVDPV2 cases [23]. However, Figure 2 illustrates a drastic shift in the prevalence of WPV1 and cVDPV2 cases beginning in 2020. Likewise, between 2021 and 2023, there are few, if any, WPV1 cases, which contradicts the large number of cases reported prior to the pandemic [23]. Inconsistencies in reporting likely contribute to the general trends observed, yet the increased prevalence of cVDPV cases is alarming considering the mass proportion of children that were not immunized for polio during the COVID-19 pandemic. This increase in so-called vaccine-derived polio may further existing vaccine hesitancy and make it more challenging for missed polio immunizations to be accounted for in the future.

Prior to the COVID-19 pandemic, Pakistan was making considerable progress toward polio eradication as the political push and financial assistance for polio was much greater than for other routine immunizations [6]. In general, polio received more attention and funding because eradication is a key objective for many global health organizations, including GAVI, but this meant other routine immunizations were often neglected. A door-to-door micro census displayed how oftentimes children received all polio doses but missed routine immunizations for other VPDs because they were not promoted in the same manner [6]. However, there was also often resistance to polio vaccinations as many individuals believed vaccinations served as a political initiative rather than a public health motive. For instance, a fake hepatitis B vaccination campaign held in 2011 to track Osama Bin Laden fueled mistrust of immunization activities [24], and vaccine hesitancy due to political and personal beliefs persisted during and after the COVID-19 pandemic. After polio immunization activities were resumed in July 2020, a study in Sindh Providence found that many refused to vaccinate their children for polio because they claimed their children were regularly vaccinated yet showed no improvements in their health [25]. As a result of poor education, lack of accessibility to health information, and rising surges of misinformation, individuals were doubtful of vaccine initiatives, especially following a shift in priorities from only polio to COVID-19. Despite this, Pakistan may be more likely to recover polio losses because of the disease’s high priority in funding and administrative efforts by GAVI and the EPI, whereas Pakistan may continue to struggle with more neglected VPDs.

Impact of COVID-19 on tuberculosis

**Figure 3:**
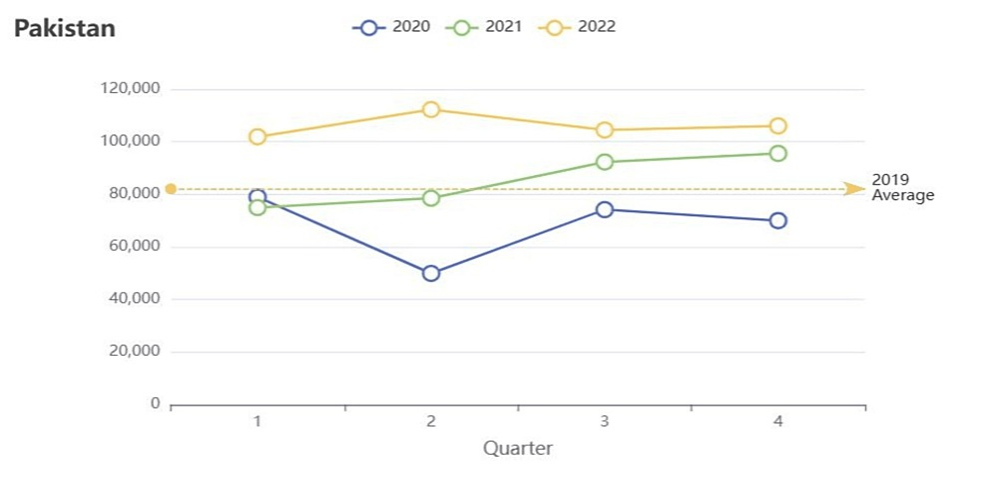
Provisional number of people with new or relapse episodes of TB notified per quarter *Reproduced from “Provisional tuberculosis (TB) notifications: Pakistan” by World Health Organization [28], Copyright 2024. Reprinted with permission.

As a result of overwhelmed health facilities and resources, the COVID-19 pandemic had a significant impact on routine immunization and the prevalence of tuberculosis in Pakistan. Accounting for nearly 61% of the global tuberculosis burden, Pakistan has approximately 510,000 new cases of tuberculosis every year, many of which are from drug-resistant strains [26]. During the COVID-19 pandemic, Pakistan experienced a 66.8% decline in Bacillus Calmette-Guerin (BCG) vaccination as many tuberculosis health services were limited due to a shortage of healthcare workers and hospitals, and the conversion of tuberculosis laboratories into COVID-19 testing centers [27]. Due to disruptions by the pandemic, essential tuberculosis activities were often neglected for diseases of higher priority like COVID-19 and even polio. That said, Figure 3 serves to help recognize the potential impacts the COVID-19 pandemic may have had on tuberculosis notifications from 2020 to 2022, with the average case notifications in 2019 serving as a baseline [28]. A significant decline in case notifications is observable between the first two quarters of 2020, which corresponds to the beginning of the COVID-19 pandemic. Likewise, there are multiple factors that may have influenced the steep decline in notifications in 2020. For example, since they share similar symptoms as a pulmonary respiratory disease [27], individuals with tuberculosis may have been misdiagnosed with COVID-19, meaning the true extent of tuberculosis during the pandemic may not be fully understood. Additionally, individuals possibly infected with tuberculosis may not have sought testing due to a fear of being stigmatized or socially isolated from the community because of COVID-19 stigmas [27]. However, also to be noted in Figure 3 is that the steep decline in case notifications in 2020 is quickly followed by a sharp spike in notifications and continues to follow a general increasing pattern through 2022 [28]. Due to this, it is likely tuberculosis testing will increase in 2021 and 2022, but it cannot be assumed that these notifications are truly representative of the tuberculosis burden in Pakistan. Furthermore, given the pandemic had exhausted the country of its funding and resources, likely, disruptions in tuberculosis services were not immediately resumed, and many cases may have been underreported or misdiagnosed. Figure 3 displays approximately 110,000 case notifications in 2022, while the WHO estimates a tuberculosis incidence between 447,000 and 826,000 [28,29]. Moreover, this incredible gap between the incidence and notified cases indicates that Pakistan may be struggling with resuming tuberculosis screening services and maintaining adequate surveillance among its provinces. Given this, addressing the shortage of healthcare workers, increasing accessibility of tuberculosis health services, and improving the consistency of surveillance of tuberculosis across the country could help narrow the gap between the incidence and the number of cases notified. Yet, a lack of proper funding and prioritization of tuberculosis remain obstacles to improving Pakistan’s tuberculosis response following the COVID-19 pandemic.

Although improved training of healthcare professionals and increased accessibility of health information to individuals could have limited the number of misdiagnoses, the disruption of routine immunization of BCG vaccines may not have been as easily addressed. Since the first dose of BCG is administered shortly after birth, many children born during the pandemic may have missed their first dose due to a fear of acquiring COVID-19 from the hospital and lockdown measures limiting transportation accessibility [27]. That said, strategies such as the use of telemedicine could have helped provide health education regarding tuberculosis while also holding mothers accountable for postnatal visits for routine immunizations, but this would not have been efficient in rural or remote areas where there may be limited smartphone accessibility. All in all, since COVID-19 created great disruptions in tuberculosis case-detection services and BCG immunizations, Pakistan may not be able to achieve the initiatives set forth by the National Tuberculosis Programme (NTP) on time. The NPT strives to reduce tuberculosis prevalence in Pakistan by 50% by 2025 through improvements in tuberculosis case notifications and program operations, but following a sharp decrease in notifications since 2020, the NPT may not be able to readily recover lost progress in Pakistan [28,30,31]. The COVID-19 pandemic was a health emergency that not only increased the prevalence of other diseases within the country but also uncovered flaws within Pakistan’s routine immunization system. While the pandemic served as an unpredictable disturbance to Pakistan’s health system, other events, such as natural disasters, have highlighted how Pakistan generally lacks adequate emergency preparedness to account for disruptions in basic immunization activities.

Targets for improvement

Since the country has experienced an ongoing struggle with routine immunizations, Pakistan has established a plan to improve the population’s health status and significantly reduce childhood mortality from VPDs through a strengthened system of routine immunizations. Within Pakistan’s Vision for 2025, several major actions are noted to achieve this goal. First, Pakistan aims to expand the outreach of the LHW program [32] since it has shown to be a cost-effective approach that has garnered community support but currently lacks the capacity to account for major immunization setbacks. Expansion of the program would not only help reinforce trust with vaccinators and improve immunization coverage but would also serve to increase access to health education. This corresponds to Pakistan’s plan to improve access to health information as well [32], given many rural or underserved communities may not have adequate access to health knowledge that would help make informed health decisions and engage in healthy behaviors. Third, Pakistan’s Vision details a plan to improve its disease surveillance system, which would help decrease inconsistencies in case reporting and make it easier to understand the areas that were most impacted by a disaster or health emergency. Lastly, Pakistan intends to develop a national plan for vaccinations [32], which may allow the country to recognize and recover from immunization setbacks more efficiently. Since decentralization of its government, Pakistani provinces may not have similar vaccination schedules, resulting in inconsistent case reporting and delivery of vaccines. Moreover, utilizing a singular plan for vaccinations may help Pakistan regain some of its structural integrity that was lost after the dissolution of the Federal Ministry of Health without compromising the authority of provinces in health planning.

Limitations

There are a few limitations to this review. First, the proposed vaccine trends are observation-based from surveillance data produced by the CDC and WHO, but there are limitations to what information is readily available from Pakistan, meaning additional data may influence the trends. Second, it is assumed that the major events mentioned corresponded with changes in immunization coverage, but other factors like internal conflict, migration, and political influence on vaccines could have contributed to changes in trends as well. Third, the observations are based on trends after the decentralization of Pakistan’s government, meaning the country’s routine immunization progress is not fully understood. Surveillance data should be collected over a longer timeframe to see the evolution of these trends prior to decentralization in order to develop a greater understanding of how health emergencies have impacted routine immunization progress over time.

## Conclusions

Since the inception of the EPI, Pakistan has made significant strides in reducing childhood morbidity and mortality due to VPDs, but further efforts should focus on improving the delivery of routine immunizations during times of need to prevent Pakistan from losing immunization progress from the last few decades. The COVID-19 pandemic in particular had an incredible impact on the immunization coverage of several VPDs, such as polio and tuberculosis, and it displayed Pakistan’s poor prioritization and emergency preparedness during multiple health crises. Similarly, floods displayed upward disease trends as Pakistan responded to issues reactively with fragmented service delivery and insufficient coverage rather than proactively planning for predictable events. If Pakistan embraced its decentralized system through better delegation of duties for case detection and the provision of routine immunization services, disease outbreaks following these disasters may not have been as severe. Pakistan is on the verge of becoming overwhelmed by preventable disease outbreaks, and if the country is to reduce vaccine-preventable deaths through improved immunization coverage, it must focus on strengthening routine immunization into an integrated system that is prepared to manage emergencies.
